# Risk factors for hyponatremia in acute exacerbation chronic obstructive pulmonary disease (AECOPD): a multicenter cross-sectional study

**DOI:** 10.1186/s12890-023-02328-4

**Published:** 2023-01-28

**Authors:** Min Xiao, Xiaoyu Wang, Hanchao Wang, Fawang Du, Yu Yao, Xiaochuan Wang, Jiajia Wang, Juan Yang, Wei Xiong, Qin Wang, Xubin Ren, Tao Zhu

**Affiliations:** 1 Respiratory Medicine and Critical Care Medicine, West China Hospital of Sichuan University, Chengdu, 610041 China; 2 Otolaryngology Head and Neck Surgery, West China Hospital of Sichuan University, Chengdu, 610041 China; 3 Respiratory Medicine and Critical Care Medicine, and Preclinical Research Center, Suining Central Hospital, Suining, 629000 Sichuan China; 4 Rheumatology Medicine, Second Affiliated Hospital of Chongqing Medical University, Chongqing, 400010 China; 5 Division of General Internal Medicine, Mayo Clinic, Rochester, MN 55905 USA; 6 Respiratory Medicine and Critical Care Medicine, Second Affiliated Hospital of Chongqing Medical University, Chongqing, 400010 China; 7 Respiratory Medicine and Critical Care Medicine, Chengdu First People’s Hospital, Chengdu, 610041 Sichuan China

**Keywords:** Acute exacerbation chronic obstructive pulmonary disease (AECOPD), Hyponatremia, Risk factors, Least absolute shrinkage and selection operator (LASSO) regression, Nomogram

## Abstract

**Background:**

Hyponatremia is an independent predictor of poor prognosis, including increased mortality and readmission, in COPD patients. Identifying modifiable etiologies of hyponatremia may help reduce adverse events in patients with AECOPD. Therefore, the aim of this study was to explore the risk factors and underlying etiologies of hyponatremia in AECOPD patients.

**Methods:**

A total of 586 AECOPD patients were enrolled in this multicenter cross-sectional study. Finally, 323 had normonatremia, and 90 had hyponatremia. Demographics, underlying diseases, comorbidities, symptoms, and laboratory data were collected. The least absolute shrinkage and selection operator (LASSO) regression was used to select potential risk factors, which were substituted into binary logistic regression to identify independent risk factors. Nomogram was built to visualize and validate binary logistics regression model.

**Results:**

Nine potential hyponatremia-associated variables were selected by LASSO regression. Subsequently, a binary logistic regression model identified that smoking status, rate of community-acquired pneumonia (CAP), anion gap (AG), erythrocyte sedimentation rate (ESR), and serum magnesium (Mg^2+^) were independent variables of hyponatremia in AECOPD patients. The AUC of ROC curve of nomogram was 0.756. The DCA curve revealed that the nomogram could yielded more clinical benefits if the threshold was between 10% and 52%.

**Conclusions:**

Collectively, our results showed that smoking status, CAP, AG, ESR, and serum Mg^2+^ were independently associated with hyponatremia in AECOPD patients. Then, these findings indicate that pneumonia, metabolic acidosis, and hypomagnesemia were the underlying etiologies of hyponatremia in AECOPD patients. However, their internal connections need further exploration.

## Introduction

Hyponatremia is a very common pathophysiological conditions in people, both in general population and hospitalized patients [1, 2]. In a population-based study, including 5,179 subjects age ≥ 55 years old, Liamis et al. showed that hyponatremia (7.7%) was the most common electrolyte disorders in people in the US [3]. Meanwhile, in a retrospective study, it was found that the prevalence of hyponatremia was 3.37% in internal medicine in southeast of China [4]. Furthermore, hyponatremia was a common complication in chronic obstructive pulmonary disease (COPD) patients [5, 6]. García-Sanz et al. showed that 10.8% hospitalized acute exacerbation of COPD (AECOPD) were combined with hyponatremia [5]. Meanwhile, Chalela et al. identified that the prevalence of hyponatremia was 15.8% in AECOPD patients [6].

Mounting evidence suggests that serum Na^+^ disorders, particularly hyponatremia, were associated with poor outcomes, such as mortality, prolonged length of hospital stay (LHS), and readmission within 30-days, in patients with different illnesses, including bone fractures, injuries, heart failure, and COPD, etc. [7–10]. In a retrospective study on surgically treated hip fracture patients, Madsen et al. showed that compared with patients without serum Na^+^ disorders (9.6%), the 30-day mortality was significantly increased in patients with hyponatremia (12.2%) [7]. It was reported that on-admission hyponatremia was an independent risk factor for all-cause mortality and cardiovascular mortality in hospitalized patients with acute heart failure (AHF) [10]. Furthermore, hyponatremia was independently associated with in-hospital mortality, prolonged LHS, and readmission in AECOPD patients [5]. Meanwhile, Chalela et al. showed that compared with AECOPD without hyponatremia, a longer LHS, greater needs for ventilator support, and higher mortality were observed in AECOPD with hyponatremia [6].

Amount of risk factors, such as female gender, age, diabetes, cancers, infectious diseases, COPD, heart failure, renal failure, liver failure, thiazide diuretics, benzodiazepines, were independently associated with hyponatremia [1, 4, 11]. However, the risk factors for hyponatremia in AECOPD patients are still not well studied. Furthermore, identifying modifiable risk factors and etiologies of hyponatremia would potentially reduce adverse events in patients with AECOPD. Therefore, the aim of current study was to provide information on which clinical features and factors are independently associated with hyponatremia in patients with AECOPD.

## Methods

### Study design and population

This multicenter cross-sectional study was performed at Respiratory Medicine and Critical Care Medicine of Suining Central Hospital and Second Affiliated Hospital of Chongqing Medical University from January 2019 to December 2021. This study was approved by the Research Ethics Committees of the Suining Central Hospital (NO. LLSLH20220046) and Second Affiliated Hospital of Chongqing Medical University (No. 2019-23) in accordance with the Declaration of Helsinki. Written informed consent was obtained from all the patients by the responsible physician or an appropriately trained staff member. According to current clinical guidelines [12–14], medical care and treatments were provided in our study.

### Sample size determinations

According to the previous studies [6, 15, 16], the ratio of normonatremia to hyponatremia in AECOPD patients ranged from 4:1 to 6:1. Meanwhile, based on our previous studies [17, 18], samples size was determined. Briefly, a minimum of 404 participants (323 normonatremia patients and 81 hyponatremia patients) were required to detect at least a 35% difference in effect size with a power of 80%, assuming α = 0.05 and an allocation ratio of 4:1. Furthermore, 20% more patients were recruited.

### Inclusion and exclusion criteria

**Fig. 1:**
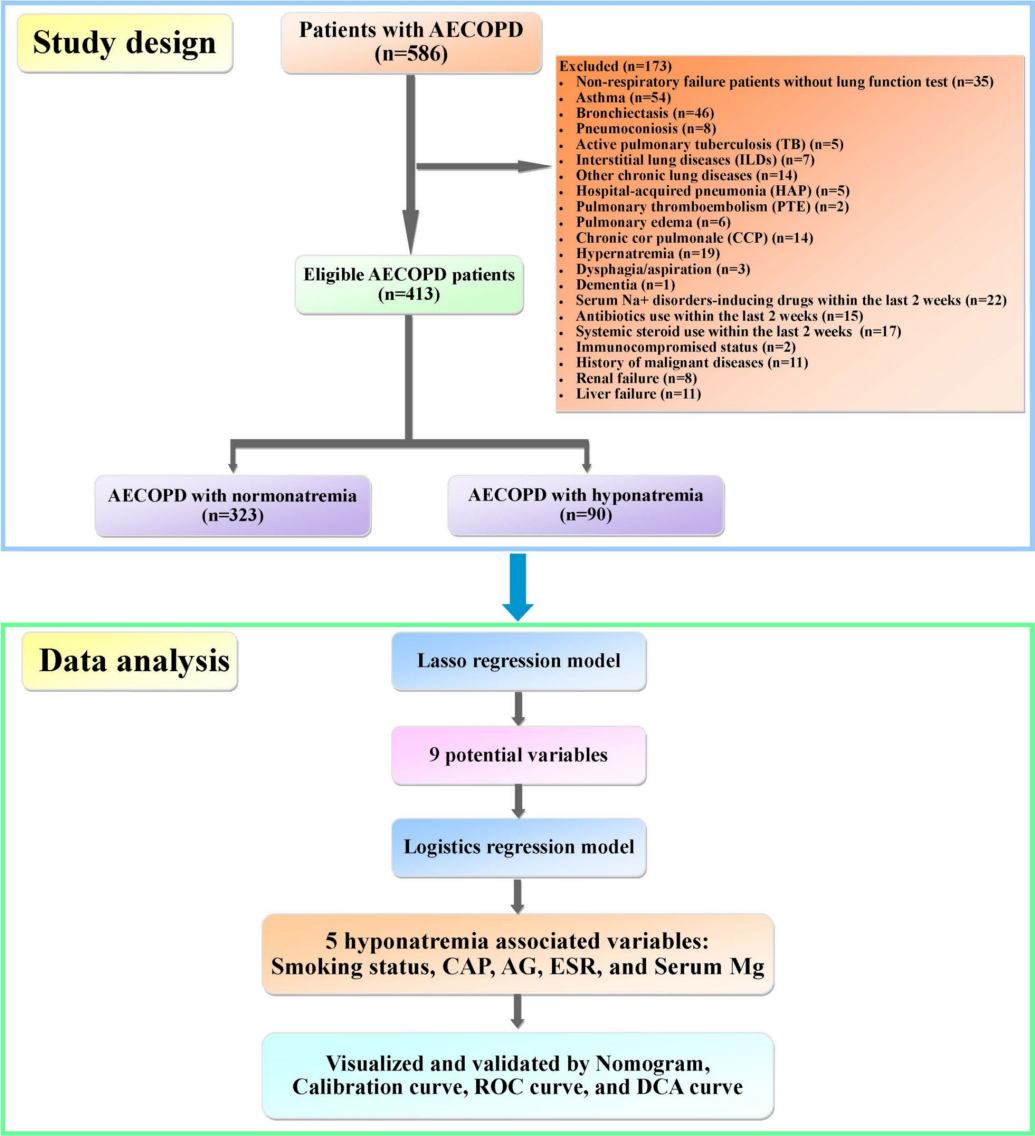
The flow diagram of the study

The inclusion criterion was an acute exacerbation of COPD requiring hospitalization in patients with age ≥ 40 years. Exclusion criteria were as follows: non-respiratory failure patients without lung function test, asthma, bronchiectasis, pneumoconiosis, active pulmonary tuberculosis (TB), interstitial lung diseases (ILDs), other chronic lung diseases, hospital-acquired pneumonia (HAP), pulmonary thromboembolism (PTE), pulmonary edema, chronic cor pulmonale (CCP), hypernatremia, dysphagia/aspiration, dementia, serum Na^+^ disorders-inducing drugs [such as diuretics, angiotensin-converting enzyme (ACE) inhibitors, angiotensin II receptor blockers (ARBs), non-steroidal anti-inflammatory drugs (NSAIDs), proton-pump inhibitors (PPIs), etc.] within the last 2 weeks, system steroid use within the last 2 weeks, immunocompromised status (organ transplant and immunosuppressive agents use), history of malignant diseases, renal failure, and liver failure. A total of 586 patients with hospitalized AECOPD were enrolled. And 173 were excluded. To the end, 323 had normonatremia (serum sodium 135–145 mmol/L), and 90 had hyponatremia (serum sodium < 135 mmol/L) (Fig. 1).

### Definitions

According to GOLD guideline, the diagnosis of COPD was confirmed by the pulmonologists, based on noxious stimuli exposure history, risk factors, clinical symptoms, and spirometry (FEV1/FVC% < 0.7 after bronchodilator inhalation) [12]. AECOPD was defined as an event in the natural course of the disease characterized by acute changes in clinical symptoms beyond normal day-to-day variation, resulting in additional therapy [12, 19]. Normonatremia was defined as serum Na^+^ 135–145 mmol/L [1, 20]. Hyponatremia was defined as serum Na^+^ < 135 mmol/L [1, 20]. Community-acquired pneumonia (CAP) was defined by the typical symptoms and signs of systemic or acute lower respiratory tract infection and with evidence compatible with a diagnosis of CAP on chest computed tomography (CT) [17, 21]. Participants were defined as ex-smokers if they had abstained from smoking for ≥ 6 months [17, 18]. The neutrophil-to-lymphocyte ratio (NLR) was defined as the number of neutrophils divided by the lymphocytes in the blood [17, 18].

### Data collection

Demographic data, underlying diseases, comorbid conditions, symptoms, arterial blood gas (ABG), serum electrolytes, liver functions, renal functions, and inflammatory parameters were recorded and collected. The blood samples for laboratory tests and lung function tests were all collected and performed within 24 h after admission. However, for the safety reason and compliance concerns, spirometry test was not performed in patients with respiratory failure. All patients underwent chest HRCT scans within 48 h of admission and the images were reviewed by a radiologist and a pulmonologist independently in each hospital.

### Statistical analysis

Data were analyzed using R software version 4.1.2.  The distribution of continuous variables was analyzed by Kolmogorov–Smirnov test. Continuous variables with normal distribution were expressed as the Mean ± standard deviation (SD), continuous variables with abnormal distribution were presented as the Median and interquartile range [Mean (Q1, Q3)], and categorical data were expressed as the frequencies. Continuous variables with abnormal distribution and ordinal variables were measured by Mann–Whitney U-test. Chi square test was used to analyze categorical variables. Variable multicollinearities were assessed using variance inflation factor (VIF). Variables with VIF ≥ 10 indicated high multicollinearity, which were excluded in following analysis. The least absolute shrinkage and selection operator (LASSO) regression was used to screen potential variables associated with hyponatremia. Subsequently, based on LASSO regression-selected variables, binary logistic regression was performed to investigate the independent variables associated with hyponatremia in patients with AECOPD. Additionally, nomogram, calibration curve, receiver operating characteristic (ROC) curve, and decision curve analysis (DCA) curve were used to visualize and validate the logistic regression model (Fig. 1). Statistical significance was set at *p* < 0.05.

## Results

### Baseline characteristics of AECOPD patients

This cross-sectional study enrolled 586 patients with AECOPD. Ultimately, 323 (78.2%) patients had normonatremia, 90 (21.8%) patients had hyponatremia (Fig. 1). Among them, 189 (45.76%) patients had CAP. The ratio of hyponatremia in AECOPD was 3.59. The demographic data of the patients are shown in Table 1. AECOPD patients with hyponatremia had less smoking, and higher rates of pleural effusion and CAP.

**Table 1:** Demographic data of patients with AECOPD (n = 413)

Characteristics	Normonatremia (n = 323)	Hyponatremia (n = 90)	Statistical values	*P* value
Age (years)	70.62 ± 9.06	72.02 ± 8.82	− 1.30	0.193
Sex [Male n (%)]	251 (77.7%)	69 (76.7%)	0.04	0.834
Body mass index (BMI)	22.25 ± 3.38	22.22 ± 3.61	0.06	0.954
Smoking			− 2.19	0.029
Non-smoking	108 (33.4%)	41 (45.6%)		
Ex-smoking	76 (23.5%)	20 (22.2%)		
Current-smoking	139 (43.1%)	29 (32.2%)		
GOLD stages			− 1.15	0.251
Stage I: mild (≥ 80%)	24 (7.4%)	10 (11.1%)		
Stage II: moderate (50–79%)	115 (35.6%)	23 (25.5%)		
Stage III: severe (30–49%)	94 (29.1%)	25 (27.8%)		
Stage IV: very severe (< 30%) without respiratory failure	26 (8.1%)	7 (7.8%)		
Respiratory failure	64 (19.8%)	25 (27.8%)		
Underlying diseases/co-morbidities				
Pleural effusion [n, (%)]	7 (2.2%)	9 (10.0%)	11.60	0.001
CAP [n, (%)]	124 (38.4%)	65 (72.2%)	32.46	0.000
CAD [n, (%)]	56 (17.3%)	22 (24.4%)	2.32	0.128
Hypertension [n, (%)]	124 (38.4%)	38 (42.2%)	0.43	0.510
T2DM [n, (%)]	49 (15.2%)	19 (21.1%)	1.81	0.179
Af [n, (%)]	11 (3.4%)	8 (8.9%)	4.82	0.028
CTD [n, (%)]	2 (0.6%)	1 (1.1%)	0.24	0.627
Pneumothorax [n, (%)]	2 (0.6%)	2 (2.2%)	1.89	0.170
Mechanical ventilation (MV)			− 1.89	0.058
Non-ventilation [n, (%)]	300 (92.9%)	78 (86.7%)		
NIPPV [n, (%)]	23 (7.1%)	11 (12.2%)		
IPPV [n, (%)]	0 (0.0%)	1 (1.1%)		

*AECOPD* Acute exacerbation chronic obstructive pulmonary disease, *BMI* Body mass index, *CAP* Community-acquired pneumonia, *CAD* Coronary artery disease, *T2DM* Type 2 diabetes mellitus, *Af* Atrial fibrillation, *CTD* Connective tissue disease, *NIPPV* Non-invasive positive pressure ventilation, *IPPV* Invasive positive pressure ventilation

### Clinical presentations and laboratory data of AECOPD patients

Compared to AECOPD patients with normonatremia, the rate of fever, WBC, NS, NS%, NLR, PCT, ESR, PH, and AG were markedly higher, meanwhile, LYM, EOS, LYM%, EOS%, serum Ca^2+^, serum Mg^2+^, and ALB were significantly lower in AECOPD patients with hyponatremia (Table 2).

**Table 2:** Clinical features and laboratory data of patients with AECOPD (n = 413)

	Normonatremia (n = 323)	Hyponatremia (n = 90)	Statistical values	*P* value
Fever [n, (%)]	21 (6.5%)	13 (14.4%)	5.88	0.015
WBC (× 10^9^/L)	7.53 ± 3.35	8.36 ± 4.07	− 1.99	0.047
NS (× 10^9^/L)*	4.96 (3.58, 7.42)	5.6 (4.21, 8.03)	− 2.20	0.028
LYM (× 10^9^/L)	1.47 ± 0.81	1.26 ± 0.56	2.24	0.026
EOS (× 10^9^/L)*	0.16 (0.07, 0.31)	0.1 (0.03, 0.26)	− 2.21	0.027
NS%	74.82 ± 11.59	78.94 ± 10.72	− 3.03	0.003
LYM%*	20.59 (14.36, 29.09)	18.19 (10.77, 22.62)	− 3.14	0.002
EOS%*	2.48 (0.86, 5.09)	1.66 (0.33, 3.82)	− 2.31	0.021
NLR*	3.64 (2.31, 5.66)	4.31 (3.33, 8.15)	− 3.09	0.002
RBC (× 10^12^/L)	4.47 ± 0.61	4.36 ± 0.68	1.50	0.136
HB (g/L)	133.89 ± 15.83	130.82 ± 18.53	1.57	0.118
HCT (L/L)	40.73 ± 4.64	40.03 ± 5.25	1.24	0.217
PLT (× 10^9^/L)	198.65 ± 69.98	208.21 ± 79.46	− 1.11	0.267
PCT (ng/ml)*	0.05 (0.03, 0.08)	0.07 (0.04, 0.15)	− 4.54	0.000
CRP (mg/ml)	22.08 ± 35.74	26.77 ± 38.68	− 1.08	0.280
ESR (mm/first hour)	21.67 ± 17.51	34.17 ± 23.45	− 5.52	0.000
*ABG*				
PH*	7.43 (7.41, 7.45)	7.45 (7.42, 7.47)	− 2.62	0.009
PaCO2 (mmHg)*	40 (37, 46)	38.5 (34, 46)	− 1.73	0.083
PaO2 (mmHg)	75.91 ± 15.11	75.89 ± 16.04	0.01	0.992
AB (mmol/L)	28.11 ± 5.14	28.05 ± 5.62	0.10	0.920
SB (mmol/L)	27.39 ± 3.00	27.56 ± 3.25	− 0.46	0.648
AG	10.94 ± 4.89	12.14 ± 4.71	− 2.08	0.039
Serum Na^+^ (mmol/L)*	139.60 (137.90, 141.45)	131.75 (129.43, 133.57)	− 14.52	0.000
Serum K^+^ (mmol/L)	3.95 ± 0.41	3.93 ± 0.54	− 0.68	0.500
Serum Ca^2+^ (mmol/L)	2.25 ± 0.14	2.21 ± 0.18	2.24	0.026
Serum Mg^2+^ (mmol/L)	0.86 ± 0.11	0.83 ± 0.09	2.94	0.003
ALB (g/L)*	38.50 (36.10, 41.10)	36.9 (33.65, 40.25)	− 2.80	0.005
BUN (mmol/L)	6.44 ± 2.31	6.36 ± 2.66	0.26	0.793
Cr (μmol/L)	73.32 ± 21.11	72.57 ± 30.93	0.27	0.790
ALT (U/L)	22.38 ± 21.55	20.86 ± 18.85	0.61	0.542
AST (U/L)	23.94 ± 19.80	24.78 ± 19.95	− 0.36	0.719
TBIL (μmol/L)	10.58 ± 4.89	11.71 ± 6.09	− 1.84	0.067
IBIL (μmol/L)	6.13 ± 3.14	6.69 ± 3.57	− 1.45	0.148
DBIL (μmol/L)	4.45 ± 2.54	5.02 ± 3.22	− 1.77	0.077
RBG (mmol/L)	6.67 ± 2.48	7.22 ± 2.67	− 1.80	0.072

*Data presented as Median (Q1, Q3)

*AECOPD* Acute exacerbation chronic obstructive pulmonary disease, *WBC* White blood cells, NS Neutrophils, *LYM* Lymphocytes, *EOS* Eosinophils, *NLR* Neutrophil–lymphocyte ratio, *RBC* Red blood cell, HB Hemoglobin, *HCT* Hematocrit, *PLT* Platelet, *PCT* Procalcitonin, *CRP* C-reactive protein, *ESR* Erythrocyte sedimentation rate, *ABG* Arterial blood gas, *AB* Actual standard bicarbonate, *SB* Standard bicarbonate, *AG* Anion gap, *Na*^+^ Sodium ions, *K*^+^ Potassium ions, *Ca*^2+^ Calcium ions, *Mg*^2+^ Magnesium ions, *ALB* Albumin, *BUN* Blood urea nitrogen, *Cr* Creatinine, *ALT* Alanine aminotransferase, *AST* Aspartate aminotransferase, *TBIL* Total bilirubin, *IBIL* Indirect bilirubin, *DBIL* Direct bilirubin, *RBG* Random blood glucose

### LASSO regression analysis of potential variables associated with hyponatremia in patients with AECOPD

**Fig. 2:**
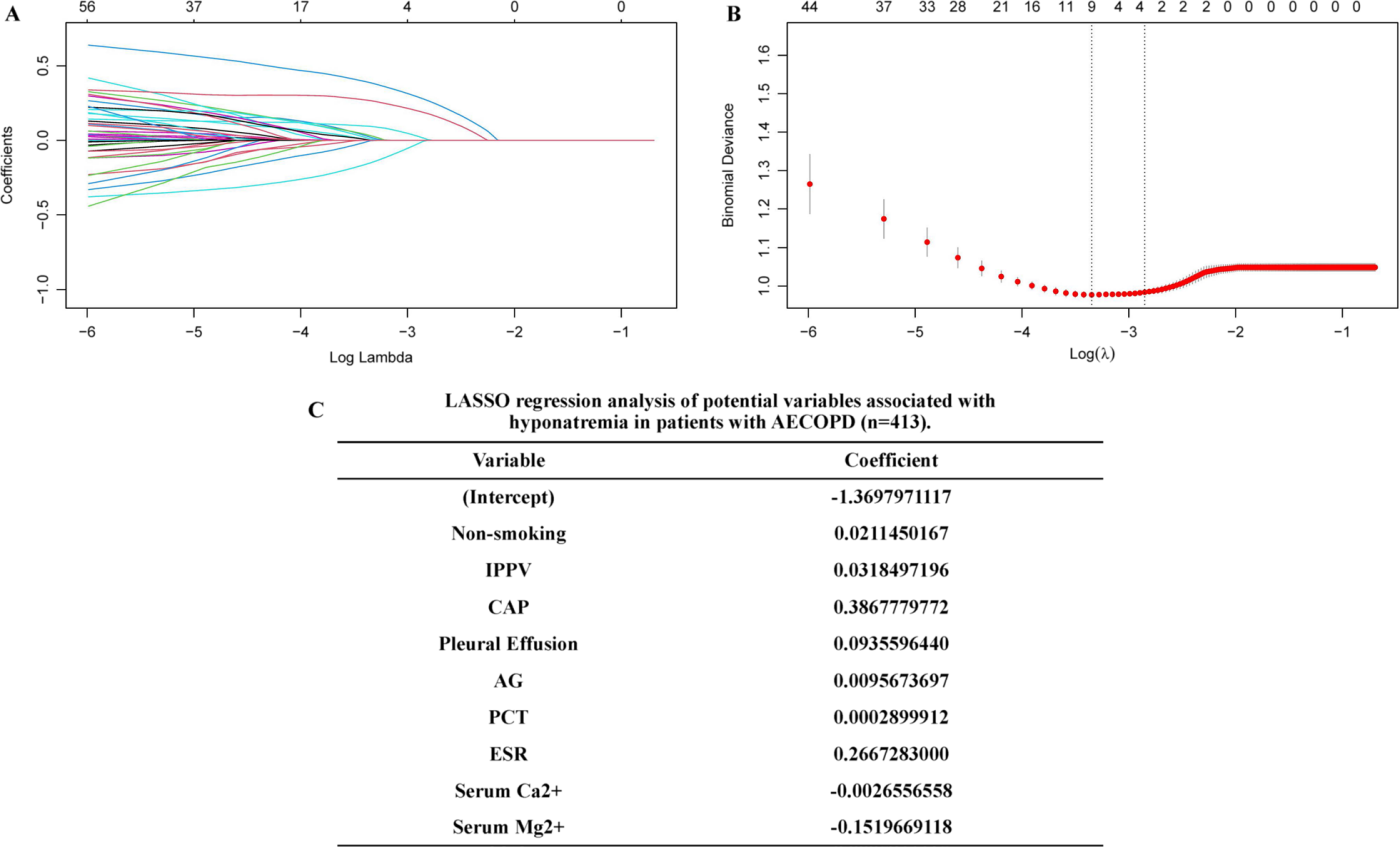
Potential variables associated with hyponatremia in AECOPD patients were selected by LASSO regression model. **A** LASSO coefficients profiles for all variables. **B** Identification of the optimal penalization coefficient (λ) in the LASSO model, which was carried out by tenfold cross-validation by minimum criteria and 1-SE (standard error) criteria. Left line: the minimum error; Right line: the cross-validated error within 1 standard error of the minimum. **C** LASSO coefficient values of 9 potential variables

LASSO regression, a shrinkage estimation method, was used to reduce data dimension and select the potential predictors, which were associated with hyponatremia in AECOPD patients. Then, 9 variables with non-zero coefficients, including non-smoking, IPPV, CAP, pleural effusion, AG, PCT, ESR, serum Ca^2+^, and serum Mg^2+^, were identified (Fig. 2).

### Binary logistic regression analysis of variables independently associated with hyponatremia in AECOPD patients

Subsequently, binary logistic regression model was established using 9 LASSO regression-selected potential variables. Then, our data revealed that smoking status (ex-smoking), the rate of CAP, AG, ESR and serum Mg^2+^ were independently associated with hyponatremia in AECOPD patients (Table 3).

**Table 3:** Binary logistics regression analysis of independent variables associated with hyponatremia in patients with AECOPD (n = 413)

	OR	OR 95% CI	*P* value
(Intercept)	58.410	0.595–5730.099	0.082
Ex-smoking	0.477	0.234–0.971	0.041
Current-smoking	0.630	0.346–1.148	0.131
NIPPV	1.272	0.525–3.085	0.594
IPPV	21,108,070.000	0.000–Infinite	0.985
CAP	2.202	0.678–7.150	0.000
Pleural effusion	1.276	0.864–1.886	0.189
AG	0.208	0.034–1.264	0.039
PCT	3.458	1.948–6.141	0.221
ESR	1.020	1.008–1.033	0.002
Serum Ca^2+^	1.061	1.003–1.122	0.088
Serum Mg^2+^	0.015	0.001–0.288	0.005

*AECOPD* Acute exacerbation chronic obstructive pulmonary disease, *CAP* Community-acquired pneumonia, *NIPPV* Non-invasive positive pressure ventilation, *IPPV* Invasive positive pressure ventilation, *AG* Anion gap, *Ca*^2+^ Calcium ions, *Mg*^2+^ Magnesium ions, *PCT* Procalcitonin, *ESR* Erythrocyte sedimentation rate, *OR* Odds ratio, *CI* Confidence interval

### Nomogram was used to visualize and validate the binary logistics regression model

**Fig. 3:**
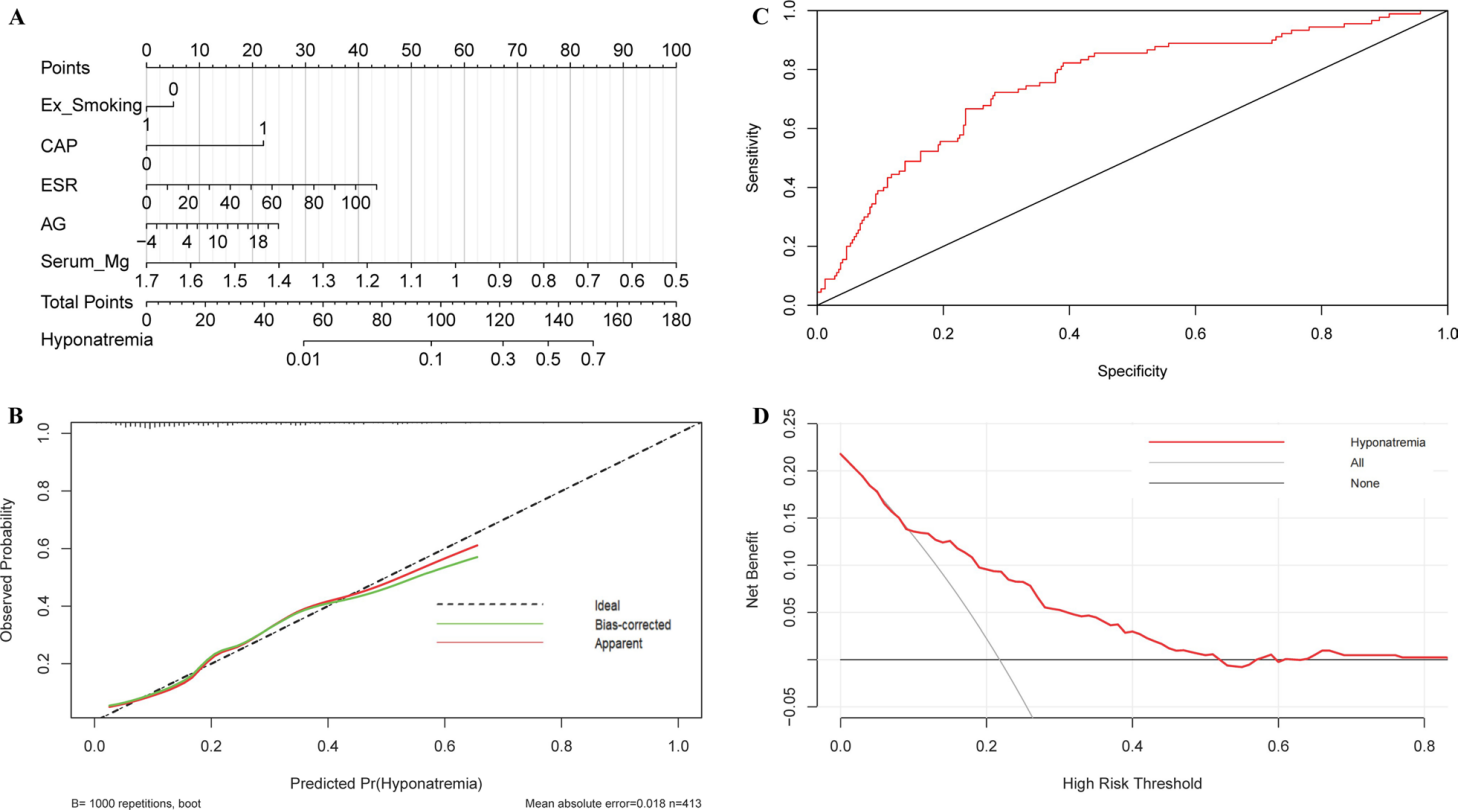
The nomogram for predicting hyponatremia in AECOPD patients. Nomogram was used to visualize and validate the binary logistics regression model. **A** Nomogram. The total point of a specific patient is the sum of individual variable points. The predicted probability of hyponatremia is on the Hyponatremia-scale, which is corresponding to Total points-scale. **B** Calibration curves. Ideal line: the nomogram reference line; Apparent line: the actual probability of each patient in our study; Bias-corrected line is adjusted by bootstrap with 1000 resamples. The length of the vertical lines at the top of the plot represents the numbers of patients. **C** ROC curve. **D** DCA curve. None line: the assumption that all patients had no hyponatremia. All line: the assumption that all patients had hyponatremia. Red line: the nomogram

Based on our established binary logistics regression model, nomogram was built. The total point of a specific patient is the sum of individual variable points (Fig. 3A). Calibration curve with 1000 bootstrap showed that both Apparent line and Bias-corrected line were close to Ideal line with mean absolute error (MAE) = 0.018 (Fig. 3B). AUC of ROC curve was 0.756 (95% CI 0.699–0.813) (Fig. 3C). The DCA curve revealed that when the threshold ranged 10% to 52%, using our nomogram to predict the hyponatremia probability yielded more net benefit than the scheme, indicating well clinical applicability of this nomogram (Fig. 3D).

## Discussion

This multicenter cross-sectional study enrolled 586 AECOPD patients. In the end, 323 (78.2%) patients with normonatremia and 90 (21.8%) patients with hyponatremia were included. Demographics, underlying diseases, pulmonary function, and comprehensive laboratory findings were collected. Firstly, LASSO regression selected 9 variables, including non-smoking, IPPV, CAP, pleural effusion, AG, PCT, ESR, serum Ca^2+^, and serum Mg^2+^, which were potentially associated with hyponatremia in AECOPD patients. Subsequently, binary logistic regression model was established using these 9 variables, which identified that smoking status (ex-smoking), the rate of CAP, AG, ESR and serum Mg^2+^ were independently associated with hyponatremia in patients with AECOPD. Furthermore, nomogram and its-associated curves were used to visualized and validate logistics regression model. The AUC of ROC curve was 0.756 (95% CI 0.699–0.813). The MAE of calibration curve was 0.018. Meanwhile, DCA curve revealed that our nomogram had a good performance to predict hyponatremia, when the threshold ranged 10% to 52%. Therefore, the results of nomogram, calibration curve, ROC curve, and DCA curve verified the accuracy, reliability, and clinical values of our binary logistics regression model. Collectively, our data suggest that pneumonia, metabolic acidosis, and hypomagnesemia are the underlying etiologies and risk factors for hyponatremia in patients with AECOPD.

Electrolyte disorders are common complications in hospitalized patients, particular in critical illnesses [20, 22–25] Hyponatremia was more frequent than other electrolytes disturbances both in general population and patients [1, 2, 20]. It was reported that the prevalence of hyponatremia was 7.7% in people great than or equal to 55 years of age in the US [3]. In a population-based cross-sectional study, enrolling 14,697 adults aged ≥ 18 years, Mohan et al. showed that the prevalence of hyponatremia was about 1.72% in the US [26]. Simultaneously, Hao et al. found that the overall prevalence of hyponatremia was 17.5% in hospitalized patients in China [9]. They also noticed that only 0.26% of cases were diagnosed. Furthermore, several studies showed that the prevalence of hyponatremia was 10.8% to 22.0% in patients with COPD [5, 6, 15]. In our study, the prevalence of hyponatremia was 21.8%, which was consistently with the previous study [15]. Hyponatremia was an independent predictor of outcomes and prognosis [5, 6, 15], such as prolonged LHS, death during hospitalization, readmission within 1-month, mechanical ventilation requirements, etc., in AECOPD patients. García-Sanz et al. revealed that hyponatremia was independently associated with prolonged LHS in AECOPD patients [5]. Chalela et al. showed that compared with AECOPD patients with normonatremia, LHS, mechanical ventilation requirements, in-hospital mortality, and all-cause mortality were markedly increased in AECOPD patients with hyponatremia [6]. However, the etiologies and risk factors for hyponatremia were not well explored. Therefore, in our study, comprehensive data including demographics, underlying diseases, comorbidities, symptoms, lung function (GOLD stages), extensive laboratory results, and chest HRCT scans were recorded.

We noticed that 20 variables were significantly different between the two groups by univariate analysis (Tables 1, 2). LASSO regression is effective in declining covariance among multiple factors, which can decrease the possibility of over-fitting and remove unnecessary covariates. Then, LASSO regression can minimize the multicollinearity in variables [27, 28]. In our study, 9 potential variables were selected by LASSO regression (Fig. 2). Subsequently, among these 9 variables, binary logistics regression identified that smoking status, the rate of CAP, AG, ESR and serum Mg^2+^ were independently associated with hyponatremia in patients with AECOPD.

Hyponatremia is not rare in infectious diseases [1, 29–31], such as *Legionella pneumophila*, pulmonary tuberculosis (TB), leptospirosis, hantavirus, cryptococcosis, malaria, coronavirus disease 2019 (COVID-19), etc. In a retrospective study, Gang et al. identified that chest infection (31.94%), malignancy (10.84%), cardiac disease (6.36%), liver cirrhosis (6.07%), and neurological disease (5.20%) were the leading etiologies of hyponatremia in internal medicine patients [4]. Furthermore, several studies reported that hyponatremia was also independently associated with prognosis of CAP [22, 32]. Potasso et al. found that hyponatremia at discharge was a predictor of re-admission in hospitalized patients with CAP [32]. Ravioli et al. demonstrated that the prevalence of hyponatremia in CAP was 28.8% in emergency patients [22]. Meanwhile, they found that hyponatremia was markedly associated with longer hospital stay in CAP patients. The underlying mechanism of hyponatremia in infectious diseases are very complicated [29, 33], which entailed Syndrome of Inappropriate Antidiuretic Hormone Secretion (SIADH), hyperproteinemia, hyperlipidemia, hyperglycemia, increased catecholamines, glucagon, and cortisol, etc. Acute exacerbation is the most common cause for admission and mortality of COPD, who were with diverse risk levels, phenotypes, and clinical features [34, 35]. Concurrently, several studies revealed that CAP was a major contributor of acute exacerbation and increased the risk of mortality and readmission in COPD patients [5, 36, 37]. In a prospective cohort study in Korea, Shin et al. showed that CAP increased 180-day mortality (HR = 1.982, 95% CI 1.164–3.375) in AECOPD patients [36]. Meanwhile, in a retrospective cohort in China, it was found that CAP significantly increased in-hospital mortality (HR = 5.29, 95% CI 1.50–18.47) in patients with AECOPD [37]. Furthermore, in a prospective cohort in Spain, García-Sanz et al. identified that both pneumonia (HR = 1.84, 95% CI 1.05–3.24) and hyponatremia (HR = 3.68, 95% CI 1.57–8.58) were independently associated with readmission within 1 month in AECOPD patients [5]. They also presumed that SIADH was the major underlying mechanism.

Simultaneously, infection, particularly respiratory tract infection, is the most important risk factor of acute exacerbation of COPD [12, 18, 35]. The ESR is a commonly used parameter to evaluate the severity of inflammation and infection in clinical practice, which were increased in CAP and AECOPD [21, 38]. Furthermore, Gao et al. revealed that increased ESR was an independently predictor of CAP in patients with AECOPD [21]. Infection-induced tissue hypoperfusion and circulation failure are the major causes for hyperlactacidemia in patients without renal failure and ketoacidosis, which can lead to increased AG, even metabolic acidosis (when AG > 16). Wang et al. found that metabolic acidosis was independently linked to mortality in children with CAP [39]. Wilson et al. demonstrated that metabolic acidosis was a maker of severe CAP [40]. Consistently, our results identified that ESR, AG, and the rate CAP were independently associated with hyponatremia in patients with AECOPD. Therefore, these results indicate that CAP is a critical etiology and risk factor of hyponatremia in AECOPD patients.

Magnesium is a critical cofactor of more than 300 enzymes, involving a number of physiological functions [41], such as energy metabolism, myocardial contraction, blood pressure, muscle contraction, airway bronchodilation, mast cell stabilization, mucociliary clearance, etc. Hypomagnesemia was also associated with poor prognosis and adverse outcomes, such as frequent readmissions, increased severity of the disease, and prolonged LHS, in COPD patients [41–44]. In a prospective observational study, Gumus et al. revealed that serum magnesium level during exacerbation period was the most significant predictor of re-acute exacerbation in hospitalized patients with AECOPD [43]. Bhatt et al. showed that serum magnesium was a predictor of frequent readmissions of AECOPD patients [42]. Serum magnesium and sodium metabolisms are profoundly and mutually interacted [41, 45]. Na^+^/Mg^2+^ exchanger (NME) plays a hub role in serum sodium and magnesium homeostasis [45]. Simultaneously, magnesium is a critical cofactor for Na^+^/K^+^-ATPase, which also is driving force for NME [41]. Collectively, magnesium deficiency (hypomagnesemia) can lead to increase in intracellular Na^+^ (decrease in extracellular Na^+^), as well as decreasing in intracellular K^+^ (increased in extracellular K^+^) [41]. Additionally, mounting evidence identified that inadequate dietary intake and its-induced malnutrition and weight loss were prevalent in COPD patients, particularly in advanced stage COPD [46], which also could account for hyponatremia and hypomagnesemia. Consistently, our data showed that hypomagnesemia was an independent predictor of hyponatremia in AECOPD patients. Otherwise, lung function [forced vital capacity (FVC) and forced expiratory volume (FEV)] was improved by dietary magnesium intake [41, 47]. Meanwhile, magnesium supplement was a promising and potential treatment for COPD and asthma [48, 49]. Further studies with more focus on the role of magnesium on the prognosis and treatment of COPD is therefore suggested.

Additionally, we also identified that ex-smoking (OR = 0.477) was independently associated with hyponatremia in AECOPD patients, suggesting that quitting smoking reduces the probability of hyponatremia. However, the association between smoking and hyponatremia and other electrolyte disorders remain unanswered at present. Future studies on this topic are therefore recommended.

Subsequently, a nomogram was established to visualize and validate our binary logistics regression model. Our data showed that AUC of ROC curve was 0.756. Ideal line and Apparent line were very closed in calibration curve, with MAE = 0.018. These results suggest the high accuracy and uniformity of our nomogram. Concurrently, DCA curve implies that our nomogram has good clinical practical value. Meanwhile, nomogram also showed that the contribution of ex-smoking was smallest and serum magnesium was biggest to hyponatremia in patients with AECOPD, indicating a strong association between serum sodium and serum magnesium. Therefore, it was necessary and critical to correct hyponatremia and hypomagnesemia simultaneously. Moreover, we presumed that appropriate magnesium supplementary intake could potentially prevent and help to correct hyponatremia in COPD patients. However, further studies should be carried out to validate this hypothesis.

In our study, comprehensive data, including demographics, underlying diseases, comorbidities, symptoms, lung function, and laboratory data, were collected, which was one of major strengths of our study. Chest HRCT scan was performed in each patient, which was useful to exclude other lung diseases and to identify the co-morbidities and complications of AECOPD, reducing confounders and promoting the accuracy of our results. Furthermore, LASSO regression was performed to reduce the multicollinearity in variables and select potential hyponatremia-associated variables, which is more accurate than univariate analysis. Meanwhile, nomogram was used to visualize and verify binary logistics regression model. However, owing to cross-sectional design, the associations between hyponatremia and prognosis and outcomes were not explored in AECOPD patients. The etiologies of acute exacerbation, which are potentially associated with hyponatremia, were not investigated, which was the major limitation of our study. This study was only performed in Chinese patients, which reduced confounders in analysis. However, further studies need be carried out in other ethnics and countries, making them more generalizable. Additionally, the information and data of therapies in stable phases of COPD were not included.

## Conclusions

In all, we found that smoking status (ex-smoking), CAP, AG, ESR and serum Mg^2+^ were independently associated with hyponatremia in AECOPD patients. These results indicate that infection, particularly CAP, and its-induced tissue hypoperfusion, circulation failure and metabolic acidosis are the major etiologies and risk factors of hyponatremia in patients with AECOPD. Then, smoking potentially leads to hyponatremia in AECOPD patients. However, its underlying mechanism needs further investigation. Additionally, magnesium was essential for serum sodium metabolism in AECOPD patients, implying hyponatremia and hypomagnesemia should be treated concurrently. Therefore, further studies should be implemented to explore this hypothesis in future.

## Data Availability

Due to the respect to and the protection of patient privacy, the data generated and/or analyzed in this study are not publicly available. However, they are available from the corresponding authors on reasonable request.

## References

[1] Spasovski G, Vanholder R, Allolio B, Annane D, Ball S, Bichet D, Decaux G, Fenske W, Hoorn EJ, Ichai C (2014). Clinical practice guideline on diagnosis and treatment of hyponatraemia. Eur J Endocrinol.

[2] Buffington MA, Abreo K (2016). Hyponatremia: a review. J Intensive Care Med.

[3] Liamis G, Rodenburg EM, Hofman A, Zietse R, Stricker BH, Hoorn EJ (2013). Electrolyte disorders in community subjects: prevalence and risk factors. Am J Med.

[4] Gang X, Zhang Y, Pan X, Guo W, Li Z, Wang Y, Wang G (2018). Hyponatremia: Prevalence and characteristics in internal medicine patients in southeast of China. Medicine (Baltimore).

[5] García-Sanz MT, Martínez-Gestoso S, Calvo-Álvarez U, Doval-Oubiña L, Camba-Matos S, Rábade-Castedo C, Rodríguez-García C, González-Barcala FJ (2020). Impact of hyponatremia on COPD exacerbation prognosis. J Clin Med.

[6] Chalela R, González-García JG, Chillarón JJ, Valera-Hernández L, Montoya-Rangel C, Badenes D, Mojal S, Gea J (2016). Impact of hyponatremia on mortality and morbidity in patients with COPD exacerbations. Respir Med.

[7] Madsen CM, Jantzen C, Lauritzen JB, Abrahamsen B, Jorgensen HL (2016). Hyponatremia and hypernatremia are associated with increased 30-day mortality in hip fracture patients. Osteoporos Int.

[8] Saepudin S, Ball PA, Morrissey H (2015). Hyponatremia during hospitalization and in-hospital mortality in patients hospitalized from heart failure. BMC Cardiovasc Disord.

[9] Hao J, Li Y, Zhang X, Pang C, Wang Y, Nigwekar SU, Qiu L, Chen L (2017). The prevalence and mortality of hyponatremia is seriously underestimated in Chinese general medical patients: an observational retrospective study. BMC Nephrol.

[10] Lu DY, Cheng HM, Cheng YL, Hsu PF, Huang WM, Guo CY, Yu WC, Chen CH, Sung SH (2016). Hyponatremia and worsening sodium levels are associated with long-term outcome in patients hospitalized for acute heart failure. J Am Heart Assoc.

[11] Sood N, Sharma KN, Himral P, Sharma T, Kapoor D (2020). Clinical profile of patients with hyponatremia in a tertiary care hospital in the sub-Himalayan region. J Family Med Prim Care.

[12] (GOLD). GIfCLD: global strategy for the diagnosis, management, and prevention of chronic obstructive pulmonary disease (Revised 2020). Preprint at https://goldcopd.org/gold-reports/ 2020.

[13] Lim WS, Woodhead M (2011). British Thoracic Society adult community acquired pneumonia audit 2009/10. Thorax.

[14] Bartlett JG, Dowell SF, Mandell LA, File TM, Musher DM, Fine MJ (2000). Practice guidelines for the management of community-acquired pneumonia in adults. Infectious Diseases Society of America. Clin Infect Dis.

[15] Tokgöz Akyıl F, Tural Önür S, Abalı H, Sökücü S, Özdemir C, Boyracı N, Kocaoğlu A, Altın S (2021). Hyponatremia is an independent predictor of emergency department revisits in acute exacerbation of COPD. Clin Respir J.

[16] Lindner G, Herschmann S, Funk GC, Exadaktylos AK, Gygli R, Ravioli S (2022). Sodium and potassium disorders in patients with COPD exacerbation presenting to the emergency department. BMC Emerg Med.

[17] Wang H, Yang T, Yu X, Chen Z, Ran Y, Wang J, Dai G, Deng H, Li X, Zhu T (2022). Risk factors for length of hospital stay in acute exacerbation chronic obstructive pulmonary disease: a multicenter cross-sectional study. Int J Gen Med.

[18] Dai G, Ran Y, Wang J, Chen X, Peng J, Li X, Deng H, Xiao M, Zhu T (2020). Clinical differences between eosinophilic and noneosinophilic acute exacerbation of chronic obstructive pulmonary disease: a multicenter cross-sectional study. Mediat Inflamm.

[19] Chronic Obstructive Pulmonary Disease Committee (2013). Respiratory Society CMA: guideline for diagnosis and treatment of chronic obstructive pulmonary disease (Version 2013). Chin J Tuberc Respir Dis.

[20] Spasovski G, Vanholder R, Allolio B, Annane D, Ball S, Bichet D, Decaux G, Fenske W, Hoorn EJ, Ichai C (2014). Clinical practice guideline on diagnosis and treatment of hyponatraemia. Nephrol Dial Transplant.

[21] Gao S, Duan Y, Chen J, Wang J (2021). Evaluation of blood markers at admission for predicting community acquired pneumonia in chronic obstructive pulmonary disease. COPD.

[22] Ravioli S, Gygli R, Funk GC, Exadaktylos A, Lindner G (2021). Prevalence and impact on outcome of sodium and potassium disorders in patients with community-acquired pneumonia: a retrospective analysis. Eur J Intern Med.

[23] Lindner G, Schwarz C, Haidinger M, Ravioli S (2022). Hyponatremia in the emergency department. Am J Emerg Med.

[24] Malieckal DA, Uppal NN, Ng JH, Jhaveri KD, Hirsch JS (2021). Electrolyte abnormalities in patients hospitalized with COVID-19. Clin Kidney J.

[25] Thongprayoon C, Cheungpasitporn W, Petnak T, Miao J, Qian Q (2021). Increased short-term and long-term mortality in community- and hospital-acquired hypernatraemia and in patients with delayed serum sodium correction. Int J Clin Pract.

[26] Mohan S, Gu S, Parikh A, Radhakrishnan J (2013). Prevalence of hyponatremia and association with mortality: results from NHANES. Am J Med.

[27] Deng X, Yang M, Wang S, Wang Q, Pang B, Wang K, Zhang Z, Niu W (2021). Factors associated with childhood asthma and wheeze in Chinese preschool-aged children. Front Med (Lausanne).

[28] Shi H, Xiang S, Huang X, Wang L, Hua F, Jiang X (2020). Development and validation of a nomogram for predicting the risk of obstructive sleep apnea in patients with type 2 diabetes. Ann Transl Med.

[29] Królicka AL, Kruczkowska A, Krajewska M, Kusztal MA (2020). Hyponatremia in infectious diseases—a literature review. Int J Environ Res Public Health.

[30] Moritz ML (2019). Syndrome of inappropriate antidiuresis. Pediatr Clin North Am.

[31] Lim AKH, Paramaswaran S, Jellie LJ, Junckerstorff RK (2019). A cross-sectional study of hyponatremia associated with acute central nervous system infections. J Clin Med.

[32] Potasso L, Sailer CO, Blum CA, Cesana-Nigro N, Schuetz P, Mueller B, Christ-Crain M (2020). Mild to moderate hyponatremia at discharge is associated with increased risk of recurrence in patients with community-acquired pneumonia. Eur J Intern Med.

[33] Ye Q, Peng X, Zhang X, Cao Q, Tao K, Wang L (2022). Clinical analysis of 103 cases of tuberculous meningitis complicated with hyponatremia in adults. Neurol Sci.

[34] Soler-Cataluña JJ, Miralles C (2021). Exacerbation syndrome in COPD: a paradigm shift. Arch Bronconeumol (Engl Ed).

[35] Golpe R, Figueira-Gonçalves JM, Esteban C, Amado-Diago CA, Aramburu A, García-Talavera I, Veiga I (2022). Relationship between the GesEPOC 2021 classification of risk levels and phenotypes and the incidence of adverse events. Arch Bronconeumol.

[36] Shin B, Kim SH, Yong SJ, Lee WY, Park S, Lee SJ, Lee SJ, Lee MK (2019). Early readmission and mortality in acute exacerbation of chronic obstructive pulmonary disease with community-acquired pneumonia. Chron Respir Dis.

[37] Lu Z, Cheng Y, Tu X, Chen L, Chen H, Yang J, Wang J, Zhang L (2016). Community-acquired pneumonia and survival of critically ill acute exacerbation of COPD patients in respiratory intensive care units. Int J Chron Obstruct Pulmon Dis.

[38] Taylan M, Demir M, Kaya H, Selimoglu Sen H, Abakay O, Carkanat A, Abakay A, Tanrikulu AC, Sezgi C (2017). Alterations of the neutrophil-lymphocyte ratio during the period of stable and acute exacerbation of chronic obstructive pulmonary disease patients. Clin Respir J.

[39] Wang LJ, Mu SC, Lin CH, Lin MI, Sung TC (2013). Fatal community-acquired pneumonia: 18 years in a medical center. Pediatr Neonatol.

[40] Wilson PA, Ferguson J (2005). Severe community-acquired pneumonia: an Australian perspective. Intern Med J.

[41] Al Alawi AM, Majoni SW, Falhammar H (2018). Magnesium and human health: perspectives and research directions. Int J Endocrinol.

[42] Bhatt SP, Khandelwal P, Nanda S, Stoltzfus JC, Fioravanti GT (2008). Serum magnesium is an independent predictor of frequent readmissions due to acute exacerbation of chronic obstructive pulmonary disease. Respir Med.

[43] Gumus A, Haziroglu M, Gunes Y (2014). Association of serum magnesium levels with frequency of acute exacerbations in chronic obstructive pulmonary disease: a prospective study. Pulm Med.

[44] Makwana S, Patel A, Sonagara M (2022). Correlation between serum magnesium level and acute exacerbation in patients with chronic obstructive pulmonary disease (COPD). Cureus.

[45] Gagnon KB, Delpire E (2020). Sodium transporters in human health and disease. Front Physiol.

[46] Nguyen HT, Collins PF, Pavey TG, Nguyen NV, Pham TD, Gallegos DL (2019). Nutritional status, dietary intake, and health-related quality of life in outpatients with COPD. Int J Chron Obstruct Pulmon Dis.

[47] de Baaij JH, Hoenderop JG, Bindels RJ (2015). Magnesium in man: implications for health and disease. Physiol Rev.

[48] Jahangir A, Zia Z, Niazi MRK, Sahra S, Jahangir A, Sharif MA, Chalhoub MN. Efficacy of magnesium sulfate in the chronic obstructive pulmonary disease population: a systematic review and meta-analysis. Adv Respir Med. 2022. 10.5603/ARM.a2022.0012 35099052

[49] Gross Júnior M, Lago PM, Santana JCB, Biondo GF, Zandoná B, Chiaradia FO, Carvalho PRA (2021). Use of magnesium sulfate in continuous infusion in patients with severe acute asthma, in a pediatric emergency room. Pediatr Pulmonol.

